# Post-Dental and Alveolar Nerve-Related Trigeminal Pain in Patients Referred with Trigeminal Neuralgia Terminology: A Retrospective Tertiary-Center Diagnostic Pathway Study

**DOI:** 10.3390/diagnostics16111674

**Published:** 2026-05-29

**Authors:** Shachar Zion Shemesh, Paz Kelmer, Jose Asprilla, Yotam Hadari, Itay Goor Aryeh, Lior Ungar

**Affiliations:** 1 Department of Neurosurgery, Sheba Medical Center, Ramat Gan 5266202, Israelasprilla_05@hotmail.com (J.A.); lior.ungar@sheba.health.gov.il (L.U.); 2 Gray Faculty of Medicine, Tel Aviv University, Tel Aviv 6997801, Israel; 3 Pain Center, Sheba Medical Center, Ramat Gan 5266202, Israel; 4 Dina Recanati School of Medicine, Reichman University, Herzliya 4610101, Israel

**Keywords:** trigeminal neuralgia, alveolar nerve, inferior alveolar nerve, post-traumatic trigeminal neuropathy, dental extraction, diagnostic misclassification, facial pain, trigeminal neuropathy, microvascular decompression, radiofrequency thermocoagulation

## Abstract

**Background**: Trigeminal neuralgia (TN), a facial pain disorder classically characterized by recurrent brief electric-shock-like paroxysms in one or more trigeminal divisions, frequently traverses dental pathways before specialist evaluation. Conversely, dental extraction, endodontic treatment, implant procedures, and third-molar surgery may injure the inferior alveolar, superior alveolar, mental, or lingual nerves and generate painful post-traumatic trigeminal neuropathy. We sought to define the diagnostic interface between classical TN and post-dental/alveolar nerve-related trigeminal pain in a tertiary referral cohort. **Methods**: We performed a retrospective single-center diagnostic-pathway study using a clinical dataset comprising 672 unique patients. A dental-interface trigeminal candidate cohort was assembled from aggregated patient-level source notes and adjudicated into five prespecified phenotypes: confirmed alveolar neuropathy, post-extraction neuropathic onset, odontogenic diagnostic misclassification, mixed/uncertain dental-interface pain, and clean classical TN. Extracted variables included demographics, trigeminal branch documentation, sensory deficit, dental procedure history, post-extraction onset, MRI and neurovascular conflict language, secondary structural disease, TN-directed medication exposure, invasive treatment exposure, documented outcomes, and time from first specialist documentation to first dated invasive treatment. **Results**: Among 201 dental-interface trigeminal candidates, 19 patients (9.5%) had confirmed alveolar neuropathy, 31 (15.4%) had post-extraction neuropathic onset, 20 (10.0%) represented odontogenic diagnostic misclassification, 115 (57.2%) remained mixed/uncertain, and 16 (8.0%) fulfilled a clean classical TN phenotype. Overall, 114 patients (56.7%) carried explicit TN terminology somewhere in the chart. Non-classical alveolar/post-dental syndromes comprised 70 patients (34.8%). Compared with clean classical TN, this non-classical group had higher rates of documented oral sensory deficit (38.6% vs. 0.0%, *p* = 0.002), post-extraction onset (52.9% vs. 0.0%, *p* < 0.001), extraction history (61.4% vs. 0.0%, *p* < 0.001), and secondary structural disease (22.9% vs. 0.0%, *p* = 0.035). Neurovascular conflict or vascular-loop language did not distinguish non-classical alveolar/post-dental syndromes from clean classical TN (38.6% vs. 37.5%, *p* = 1.000). **Conclusions**: A substantial minority of tertiary dental-interface trigeminal referrals represented alveolar/post-dental syndromes rather than clean classical TN, even while carrying TN labels and accumulating TN-directed treatment exposure. Post-extraction onset, lower-lip/chin or intraoral sensory change, and pain persisting despite extraction should prompt careful phenotyping before classical TN-directed escalation. The alveolar–trigeminal interface can be operationalized as a recognizable diagnostic pathway with direct implications for multidisciplinary facial-pain evaluation.

## 1. Introduction

Classical trigeminal neuralgia is traditionally defined by recurrent unilateral brief electric-shock-like pain in one or more trigeminal divisions, typically provoked by innocuous stimuli such as chewing, talking, shaving, tooth brushing, or light facial touch. Contemporary classification systems now separate classical, idiopathic, and secondary TN and recognize the clinically important subgroup with concomitant continuous pain. These distinctions are not semantic: they determine the differential diagnosis, the meaning of neuroimaging findings, the threshold for medication failure, and the appropriateness of invasive neurosurgical procedures.

The practical problem in daily facial-pain practice is that trigeminal pain often crosses disciplinary boundaries. Patients with TN may first present to dentists because paroxysms localize to teeth, gingiva, or the jaw. Conversely, dental and oral-surgical procedures may injure branches of the trigeminal nerve and produce painful post-traumatic trigeminal neuropathy. Thus, the same clinical entry point—V2/V3-dominant or tooth-focused facial pain—may represent classical TN, painful post-traumatic trigeminal neuropathy, persistent dentoalveolar pain, inferior alveolar or lingual nerve injury, secondary trigeminal disease, or a mixed syndrome [1,2,3,4,5,6,7,8,9].

This distinction is clinically important because diagnostic labels can influence escalation. Once a patient carries TN terminology, treatment may shift toward sodium-channel blockers, repeated nerve blocks, radiofrequency thermocoagulation, balloon compression, stereotactic radiosurgery, or microvascular decompression. These treatments are appropriate for selected TN phenotypes, but they are not interchangeable with the diagnostic work-up and counseling required for post-dental or alveolar nerve-related neuropathic pain [7,8,9,10,11,12,13,14,15].

Prior studies have described both directions of this interface: TN may be misinterpreted as odontogenic pain, leading to unnecessary dental procedures, while dental procedures themselves may cause painful post-traumatic trigeminal neuropathy. However, fewer studies have examined how these pathways coexist within a tertiary trigeminal pain population in which patients already carry trigeminal terminology and may have accumulated TN-directed interventions [16,17,18,19,20,21,22,23,24].

The present study, therefore, focuses on the alveolar–trigeminal interface rather than on TN in general. We sought to identify a clinically meaningful subgroup in which dental chronology, alveolar nerve territory symptoms, oral sensory disturbance, persistent pain after extraction, or explicit alveolar nerve terminology suggested that the case should not be treated as clean classical TN without further scrutiny [25,26,27,28,29,30,31,32].

## 2. Materials and Methods

### 2.1. Study Design and Setting

This retrospective single-center diagnostic-pathway study was conducted using an institutional clinical dataset from a tertiary facial-pain and neurosurgical practice environment. The source dataset contained 18,007 free-text note fragments linked to 672 unique patients. Notes included chief complaint, history of present illness, referral reason, visit summary, operative and postoperative documentation, and related free-text fields. The study was approved by the local institutional review board of Sheba Medical Center, with waiver of informed consent because of the retrospective nature of the study and use of de-identified clinical data.

### 2.2. Cohort Assembly

The source dataset consisted of 18,007 free-text clinical note fragments linked to 672 unique patients. Before screening, all note fragments were aggregated at the patient level so that each patient, rather than each note, served as the unit of analysis. The dental-interface trigeminal candidate cohort was then assembled using a two-step process.

First, patients were screened for trigeminal-region pain or trigeminal terminology, including explicit TN language, trigeminal branch notation, facial pain descriptors, neuropathic facial pain wording, or TN-directed treatment exposure. Second, within this trigeminal-region population, patients were screened for dental or alveolar pathway signals. These included extraction, root-canal treatment, implant-related work-up, third-molar surgery, dental consultation, tooth-focused pain, documented dental procedure, inferior alveolar nerve, superior alveolar nerve, mental nerve, lingual nerve, post-extraction onset, or persistent pain after dental intervention.

Patients were included in the analytic cohort when at least one dental/alveolar pathway signal occurred in the context of trigeminal-region pain, trigeminal terminology, neuropathic facial pain descriptors, trigeminal branch notation, or TN-directed treatment. Patients were excluded from the analytic cohort when dental terminology appeared only incidentally without facial/trigeminal pain relevance, when the available notes were insufficient to determine a dental-interface pathway, or when the pain syndrome was clearly unrelated to the trigeminal distribution. This process yielded 201 dental-interface trigeminal candidates.

### 2.3. Phenotype Adjudication

All candidate cases were adjudicated at the patient level into five mutually exclusive phenotypes. Initial extraction and preliminary phenotype assignment were performed using aggregated patient-level notes. Cases were then reviewed by two clinician reviewers with experience in neurosurgical and facial-pain documentation. Discrepancies, uncertain classifications, or cases with overlapping features were resolved by consensus, with senior review when needed. The final adjudicated category was based on the overall clinical chronology, sensory findings, dental history, structural disease, treatment pathway, and final diagnostic formulation rather than on any single isolated keyword.

Group A, confirmed alveolar neuropathy, required explicit chart language naming inferior alveolar, superior alveolar, mental, or lingual nerve neuropathy or injury, or a clearly alveolar nerve-related final diagnostic formulation. Group B, post-extraction neuropathic onset, required onset after extraction or another dental intervention together with neuropathic descriptors and no stronger named alveolar diagnosis. Group C, odontogenic diagnostic misclassification, required extraction or another irreversible dental procedure undertaken for pain without meaningful benefit, followed by a clinical course compatible with neuralgic or neuropathic trigeminal pain rather than odontogenic disease.

Group D, mixed/uncertain dental-interface pain, was assigned when the dental link was clinically relevant but the available text did not allow secure separation among post-traumatic trigeminal neuropathy, persistent dentoalveolar pain, secondary trigeminal pain, and TN. Examples included patients with dental evaluation plus TN terminology but incomplete chronology; patients with structural disease and concurrent dental procedures; patients with prior dental intervention but unclear timing relative to pain onset; and patients with overlapping neuropathic and neuralgic descriptors without sufficient sensory mapping. Group E, clean classical TN, required a final chart pattern favoring classical TN without post-extraction onset, without documented oral sensory deficit, and without multiple sclerosis or tumor-related disease.

To avoid circularity, variables that directly contributed to phenotype adjudication were not interpreted as independent discriminators of that same phenotype. Specifically, Group B was not defined by the binary presence of “post-extraction onset” alone, but by a documented temporal relationship between extraction or another dental intervention and neuropathic pain onset, together with neuropathic descriptors and no stronger named alveolar nerve diagnosis. The variable “post-extraction onset” was, therefore, retained as a descriptive chronology/adjudication variable and was not used for inferential comparison across phenotypes or in the focused comparison between non-classical alveolar/post-dental syndromes and clean classical TN.

### 2.4. Diagnostic Logic and Positive Case Definition

For the purposes of this study, the principal positive diagnostic finding was not the mere presence of a dental history. Prior dental evaluation is common in facial pain and, by itself, does not establish a post-dental neuropathic syndrome. We, therefore, defined a positive alveolar/post-dental diagnostic signal as the presence of at least one high-specificity feature linking pain to an alveolar or oral-sensory pathway. These features included explicit documentation of inferior alveolar, superior alveolar, mental, or lingual nerve involvement; onset of neuropathic pain after extraction or another dental intervention; persistent pain after an irreversible dental procedure performed for presumed odontogenic pain; or oral sensory deficit involving the lower lip, chin, gingiva, teeth, tongue, or intraoral mucosa.

This approach was designed to distinguish three clinically different entities that are often conflated in routine documentation. The first was confirmed or likely post-traumatic alveolar nerve neuropathy, in which the dental procedure or named alveolar nerve injury was central to the pain syndrome. The second was odontogenic diagnostic misclassification, in which the dental procedure appeared to be a consequence, rather than the cause, of neuralgic pain. The third was mixed dental-interface pain, in which dental chronology, trigeminal terminology, secondary disease, and treatment history were insufficiently specific to assign causality. Clean classical TN was intentionally defined narrowly in order to serve as a comparator group: patients in this category had a TN-compatible clinical pattern without post-extraction onset, without documented oral sensory deficit, and without secondary structural disease.

Because the study was retrospective and based on free-text documentation, the adjudication framework prioritized specificity over sensitivity. Cases were not classified as confirmed alveolar neuropathy unless the chart explicitly supported alveolar nerve involvement or a closely related final diagnostic formulation. Similarly, cases were not assigned to the post-extraction neuropathic-onset group unless the temporal relationship to extraction or dental intervention was clearly documented. This conservative structure was chosen to reduce over-attribution of facial pain to dental procedures and to preserve a clinically credible comparison with clean classical TN.

### 2.5. Data Extraction

Patient-level text was aggregated before extraction. Variables included age, sex, laterality, trigeminal branch documentation, explicit TN terminology, oral sensory deficit, neuropathic wording, post-extraction onset, extraction history, dental procedure history, dental evaluation history, MRI documentation, neurovascular conflict or vascular-loop language, multiple sclerosis, tumor-related disease, carbamazepine, oxcarbazepine, pregabalin, gabapentin, blocks or injections, radiofrequency procedures, balloon compression, microvascular decompression, stereotactic radiosurgery, charted improvement, charted complete pain relief, charted recurrence, and timing from first specialist documentation to first dated invasive treatment documented as performed.

During revision, trigeminal-division variables were re-audited to avoid conflating TN1/type 1 trigeminal neuralgia terminology with anatomical ophthalmic-division V1 involvement. TN1/type 1 terminology was treated as TN phenotype terminology and was not coded as V1 anatomical involvement.

Variables were coded as present only when explicitly documented in the aggregated patient-level record. When a variable could not be recovered from the chart, it was treated as missing rather than assumed absent. No imputation was performed. Age and sex were summarized among patients with recoverable data, and categorical variables were reported using the available denominator for each variable. Tables were revised to display denominators consistently.

### 2.6. Outcome Measures

The primary outcome was the proportion of dental-interface trigeminal candidates adjudicated as non-classical alveolar/post-dental syndromes, defined as Groups A, B, and C. Secondary outcomes included discriminating clinical features between non-classical alveolar/post-dental syndromes and clean classical TN, MRI and neurovascular conflict documentation, medication and procedural exposure, documented improvement and recurrence, and the interval from first specialist documentation to first dated invasive treatment.

### 2.7. Statistical Analysis

Continuous variables are reported as mean and standard deviation or median and interquartile range, as appropriate. Categorical variables are reported as counts and percentages using the available denominator for each variable. Comparisons across the five phenotypes were performed using non-parametric testing for continuous variables and chi-square or Fisher exact testing for categorical variables. The focused comparison of greatest clinical relevance contrasted non-classical alveolar/post-dental syndromes (Groups A+B+C) with clean classical TN (Group E).

Adjudication-defining variables were displayed descriptively but were not treated as statistically independent comparative variables. Accordingly, post-extraction onset was not assigned an inferential *p* value in the revised Table 1.

Because this was an exploratory retrospective diagnostic-pathway study, correction for multiple comparisons was considered but not applied. *p* values are, therefore, interpreted descriptively and should be viewed as hypothesis-generating rather than confirmatory. The analysis was intended to describe diagnostic pathway structure rather than establish causal treatment effects.

## 3. Results

Of 672 unique patients in the source clinical dataset, 201 met criteria for the dental-interface trigeminal candidate cohort. Age was recoverable in 189 patients (94.0%); the mean age was 55.2 years and the median age was 53 years. Sex was recoverable in 189 patients (94.0%). Explicit TN terminology appeared in the chart of 114 patients (56.7%), emphasizing that TN labeling was common even within a cohort specifically enriched for dental and alveolar pathway features.

**Figure 1:**
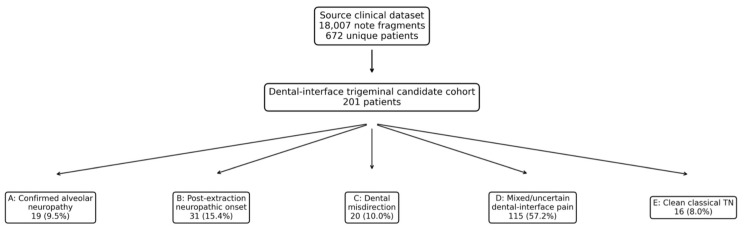
Cohort assembly and adjudicated diagnostic phenotypes. The source clinical dataset contained 18,007 note fragments linked to 672 unique patients. Screening for dental-interface trigeminal cases yielded 201 candidates, which were adjudicated into confirmed alveolar neuropathy, post-extraction neuropathic onset, odontogenic diagnostic misclassification, mixed/uncertain dental-interface pain, and clean classical TN.

The adjudicated phenotype distribution was clinically informative. A total of 19 patients (9.5%) were classified as confirmed alveolar neuropathy, 31 (15.4%) as post-extraction neuropathic onset, 20 (10.0%) as odontogenic diagnostic misclassification, 115 (57.2%) as mixed/uncertain dental-interface pain, and 16 (8.0%) as clean classical TN. Thus, non-classical alveolar/post-dental syndromes (Groups A+B+C) accounted for 70 patients (34.8%) of the dental-interface cohort, whereas clean classical TN accounted for only 16 patients (8.0%). The full cohort assembly is shown in Figure 1 and Table 2.

**Table 2:** Cohort assembly.

Metric	Value
Source clinical dataset	18,007 note fragments
Unique patients in source dataset	672
Dental-interface trigeminal candidate cohort	201 (29.9% of all unique patients)
Age recoverable within candidate cohort	189/201 (94.0%)
Sex recoverable within candidate cohort	189/201 (94.0%)
Explicit TN terminology within candidate cohort	114/201 (56.7%)
Adjudicated A+B+C phenotypes	70/201 (34.8%)
Confirmed alveolar neuropathy	19/201 (9.5%)
Post-extraction neuropathic onset	31/201 (15.4%)
Odontogenic diagnostic misclassification	20/201 (10.0%)
Mixed/uncertain	115/201 (57.2%)
Clean classical TN	16/201 (8.0%)

Groups A, B, and C together accounted for 70 patients, representing 34.8% of the dental-interface cohort. Clean classical TN accounted for 16 patients (8.0%). Explicit TN terminology appeared in 114 patients (56.7%), including many outside the clean classical TN group. This finding indicates that TN terminology in tertiary referral documentation should be interpreted as an entry point for structured phenotyping rather than as a final mechanistic diagnosis.

The mixed/uncertain group represented the largest phenotype category. These patients had clinically relevant dental-interface features but insufficient documentation to assign a single causal pathway. This group included overlapping dental, neuropathic, structural, and TN-directed treatment signals, supporting the need for more structured prospective documentation of dental chronology, sensory mapping, and response to dental intervention.

Clinical features separated the phenotypes in the expected direction. Documented oral sensory deficit was concentrated in confirmed alveolar neuropathy and post-extraction neuropathic-onset cases, occurring in 31.6% and 61.3%, respectively, compared with 0.0% of clean classical TN cases (overall *p* < 0.001). Neuropathic wording was present in 78.9% of confirmed alveolar neuropathy and 93.5% of post-extraction neuropathic-onset cases, but in only 43.8% of clean classical TN cases (*p* < 0.001). Post-extraction onset was documented in 77.4% of post-extraction neuropathic-onset cases and 50.0% of odontogenic diagnostic misclassification cases, but in none of the clean classical TN cases (*p* < 0.001).

The focused comparison between non-classical alveolar/post-dental syndromes and clean classical TN showed consistent descriptive separation across several clinical variables. Because the clean classical TN comparator group was small (n = 16), these comparisons should be interpreted cautiously and primarily as descriptive signals rather than definitive estimates of between-group effect size.

The focused comparison between non-classical alveolar/post-dental syndromes and clean classical TN showed the clearest diagnostic signal.

Because the clean classical TN comparator group was small (n = 16), these comparisons should be interpreted cautiously and primarily as descriptive signals rather than definitive estimates of between-group effect size.

Compared with clean classical TN, Groups A+B+C had higher rates of oral sensory deficit (38.6% vs. 0.0%, *p* = 0.002), extraction history (61.4% vs. 0.0%, *p* < 0.001), and secondary structural disease (22.9% vs. 0.0%, *p* = 0.035). Post-extraction onset was frequent within the non-classical alveolar/post-dental phenotypes, but because this chronology contributed to phenotype adjudication, it is shown descriptively in Table 1 and was not treated as an independent comparative variable. By contrast, neurovascular conflict or vascular-loop language did not distinguish the groups (38.6% vs. 37.5%, *p* = 1.000). This finding is diagnostically important because it indicates that imaging language compatible with neurovascular contact cannot override a clinical history and sensory map pointing toward an alveolar/post-dental phenotype (Table 1).

MRI was documented in 174 patients (86.6%). Neurovascular conflict or vascular-loop language was documented in 79 patients (39.3%) across the dental-interface cohort. Multiple sclerosis was documented in 24 patients and tumor-related disease in 44 patients, yielding secondary structural disease in 68 patients overall. Secondary structural disease was particularly common in the mixed/uncertain group, reflecting the inherent complexity of referral cases that carried both dental-interface features and additional neurological or structural explanations for trigeminal pain.

The imaging results also generated an important negative–positive finding: neurovascular conflict language was not a discriminator between non-classical alveolar/post-dental syndromes and clean classical TN. Neurovascular conflict or vascular-loop terminology appeared in 38.6% of non-classical alveolar/post-dental cases and 37.5% of clean classical TN cases. This near-identical frequency suggests that radiological contact cannot be interpreted in isolation when the clinical history points toward post-dental neuropathy. In practical terms, a vascular loop should not automatically override a history of extraction-related onset, lower-lip or chin numbness, or intraoral sensory change.

This observation is especially relevant for neurosurgical decision making. Neurovascular contact is common and may be incidental, whereas the decision to offer microvascular decompression depends on congruence between phenotype, side, trigeminal division, imaging, and expected pain mechanism. The present findings support a phenotype-first imaging interpretation strategy: MRI remains essential, but its meaning changes depending on whether the patient has clean classical TN, secondary trigeminal disease, or an alveolar/post-dental neuropathic syndrome.

Treatment exposure was substantial across all phenotypes. Carbamazepine exposure was documented in 158 patients (78.6% of the candidate cohort), pregabalin in 96 (47.8%), gabapentin in 49 (24.4%), and oxcarbazepine in 51 (25.4%). Medication exposure did not reliably distinguish non-classical alveolar/post-dental syndromes from clean classical TN, reinforcing that pharmacological TN labeling may occur before the underlying phenotype has been clarified.

**Figure 2:**
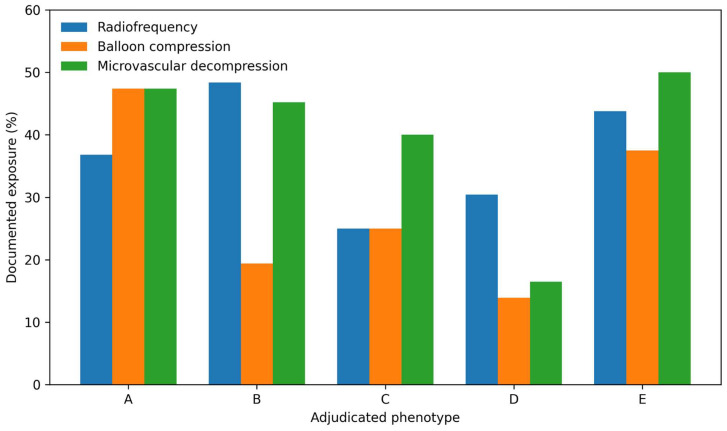
Documented exposure to radiofrequency procedures, balloon compression, and microvascular decompression by adjudicated phenotype. A = confirmed alveolar neuropathy; B = post-extraction neuropathic onset; C = odontogenic diagnostic misclassification; D = mixed/uncertain; E = clean classical TN.

Documented invasive or image-guided procedure exposure was frequent. Within the non-classical alveolar/post-dental group, blocks or injections were documented in 62 patients (88.6%), radiofrequency in 27 (38.6%), balloon compression in 20 (28.6%), microvascular decompression in 31 (44.3%), and stereotactic radiosurgery in 12 (17.1%). Procedure exposure by phenotype is shown in Table 3 and Figure 2. The most clinically provocative observation was that confirmed alveolar neuropathy and post-extraction neuropathic-onset cases accumulated exposure to TN-directed procedures, including microvascular decompression and percutaneous interventions, at rates that would not be expected if front-end phenotyping reliably separated classical TN from post-dental trigeminal neuropathy (Table 3, Figure 2).

**Figure 3:**
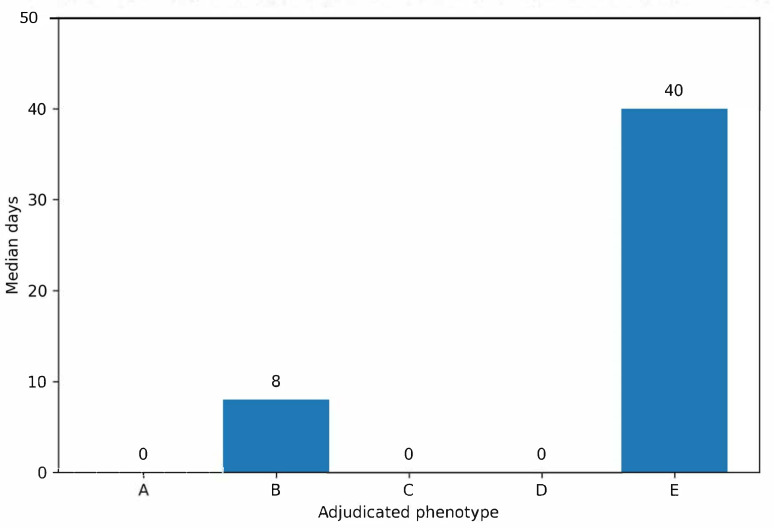
Median time from first specialist documentation to first dated invasive treatment by adjudicated phenotype. The interval represents first specialist documentation within the dataset to first invasive treatment explicitly charted as performed, not symptom onset to treatment.

Outcome documentation reflected a heavily pretreated tertiary cohort. Any improvement was documented in 160 patients (79.6%), complete pain relief in 103 (51.2%), and recurrence in 151 (75.1%). Recurrence was common across phenotypes and did not cleanly distinguish alveolar/post-dental syndromes from classical TN. First invasive treatment was dated as performed in 169 patients (84.1%). The overall median interval from first specialist documentation to first dated invasive treatment was 0 days (IQR 0–106), consistent with late referral and procedure-stage capture in many records rather than early natural-history documentation. Median intervals by phenotype are shown in Figure 3 and Table 4.

## 4. Discussion

This study identifies a recurrent diagnostic pathway vulnerability at the interface between dental medicine, neurology, pain medicine, and neurosurgery. Among tertiary dental-interface trigeminal referrals, more than one-third of cases represented confirmed alveolar neuropathy, post-extraction neuropathic onset, or odontogenic diagnostic misclassification rather than clean classical TN. At the same time, explicit TN terminology was common across the cohort, demonstrating that diagnostic labels in referral documentation may not reliably capture the underlying pain mechanism.

A central finding is that the most useful discriminators were clinical rather than radiological. The phenotype most suggestive of a non-classical alveolar/post-dental syndrome consisted of post-extraction or post-procedural onset, oral sensory deficit, neuropathic descriptors such as burning or dysesthesia, persistent pain after dental intervention, and documented inferior alveolar, superior alveolar, mental, or lingual nerve involvement. This profile is clinically distinct from clean classical TN, which is typically dominated by brief triggerable paroxysms and preserved neurological examination.

The study also illustrates a process that may be conceptualized as phenotype drift. Some patients gradually acquire TN terminology despite clinical features that are not fully consistent with classical TN, and this terminology may allow them to enter TN-oriented treatment pathways. This does not imply that TN-directed procedures are inappropriate for classical TN. Rather, it emphasizes that phenotype documentation should precede escalation, especially when the history contains post-dental onset or sensory change.

The odontogenic diagnostic misclassification group captures the opposite direction of the same interface. In some patients, irreversible dental treatment appears to have been performed for pain that later behaved more like neuralgic or neuropathic trigeminal pain than odontogenic disease. Because this study is retrospective, it cannot prove whether a dental procedure caused pain in every case. Persistent pain despite extraction should, therefore, be interpreted as a diagnostic warning sign rather than as proof of iatrogenic injury.

The published dental and oral-surgical literature indicates that post-traumatic trigeminal neuropathy is uncommon at the population level but clinically important when persistent. Reported rates vary substantially by procedure, nerve, surgical complexity, definition of neurosensory disturbance, and follow-up duration. For mandibular third-molar surgery, reported temporary inferior alveolar nerve injury rates range approximately from 0.26% to 8.4%, while permanent sensory disturbance has been reported at lower rates, up to approximately 3.6% in some series. Lingual nerve sensory disturbance is also variably reported and depends strongly on operative technique and definition. The present cohort should, therefore, not be interpreted as estimating procedure-level prevalence after dental work. Rather, it describes the phenotype composition of a tertiary referral population enriched for trigeminal and dental-interface features, in which post-dental chronology and oral sensory findings were clinically relevant to diagnostic classification.

A brief mechanistic explanation may help interpret these clinical patterns. Painful post-traumatic trigeminal neuropathy may arise after peripheral nerve injury through ectopic firing, peripheral and central sensitization, altered afferent input, and persistent sensory dysfunction in the injured nerve territory. In the alveolar–trigeminal interface, this mechanism is most relevant when pain is accompanied by lower-lip, chin, gingival, dental, tongue, or intraoral sensory disturbance after a procedure involving the inferior alveolar, mental, lingual, or superior alveolar nerves.

MRI remains essential in trigeminal pain work-up, particularly to identify secondary TN and structural lesions. However, neurovascular conflict or vascular-loop language appeared at similar rates in non-classical alveolar/post-dental syndromes and clean classical TN. This finding does not imply that neurovascular conflict is irrelevant. Instead, it reinforces that imaging should be interpreted in relation to chronology, sensory examination, pain quality, laterality, and trigeminal distribution. A vascular loop should support a TN diagnosis only when the clinical phenotype is concordant.

The proposed workflow should be interpreted as expert-informed diagnostic guidance generated from retrospective pathway analysis, not as a prospectively validated algorithm. Its practical purpose is to prevent premature closure. In patients with V2/V3-dominant, tooth-focused, or post-dental trigeminal pain, clinicians should explicitly document chronology relative to dental procedures, sensory findings in the lower lip, chin, gingiva, teeth, tongue, and intraoral mucosa, response to prior dental intervention, MRI findings, and the working phenotype before TN-directed invasive treatment is considered.

It is equally important to clarify what this study is not claiming. The findings do not imply that all post-extraction facial pain is neuropathic, that neurovascular conflict lacks relevance, or that classical TN and painful post-traumatic trigeminal neuropathy cannot coexist. The message is diagnostic rather than causal: pain in a trigeminal distribution is not, by itself, sufficient to establish mechanism.

This study has limitations. It is retrospective, single-center, and dependent on routine free-text documentation rather than a prospectively designed facial-pain registry. The clean classical TN comparator group was small, which limits statistical power and generalizability of between-group comparisons. In addition, trigeminal branch documentation in retrospective free-text notes may reflect any documented branch mention rather than dominant pain distribution, and branch-level findings were, therefore, interpreted cautiously after manual review. The candidate cohort was intentionally enriched for dental-interface cases and, therefore, should not be interpreted as estimating the population incidence of alveolar nerve injury or dental misdiagnosis among all patients with facial pain. Procedure variables reflect documented chart exposure, and the timing analysis was restricted to interventions explicitly documented as performed. Exact dates of dental procedures, symptom onset, referral, and pain recurrence were not consistently available, preventing reliable modeling of extraction-to-specialist or extraction-to-procedure intervals. In addition, neurovascular conflict was extracted from clinical language and imaging reports rather than from blinded re-review of MRI sequences. Some patients in the mixed/uncertain group may, therefore, represent true TN with dental coincidence, whereas others may represent under-documented post-traumatic neuropathy. Finally, because treatment allocation was not randomized and was strongly influenced by phenotype, patient preference, prior treatment, and referral timing, the study should not be interpreted as a comparative efficacy analysis of MVD, radiofrequency, balloon compression, stereotactic radiosurgery, or nerve blocks.

Despite these limitations, the study provides a clinically meaningful positive result. Within a large tertiary dataset, a distinct non-classical alveolar/post-dental subgroup could be identified using reproducible historical and sensory features. These patients differed from clean classical TN in post-extraction onset, oral sensory deficit, extraction history, neuropathic descriptors, and secondary structural disease, while showing similar rates of neurovascular conflict language. The resulting message is, therefore, diagnostic rather than causal: in patients with trigeminal-region pain, dental chronology and oral sensory mapping should be treated as essential parts of phenotyping before TN-directed invasive escalation.

## 5. Conclusions

A substantial subset of tertiary dental-interface trigeminal referrals represented alveolar/post-dental syndromes rather than clean classical TN. These patients often carried explicit TN terminology and accumulated TN-directed medication and invasive treatment exposure. The strongest discriminators were clinical rather than radiological: post-extraction or post-procedural onset, oral sensory deficit, persistent pain after dental intervention, neuropathic descriptors, and explicit inferior alveolar, superior alveolar, mental, or lingual nerve involvement.

The principal finding of this study is that the alveolar–trigeminal interface can be operationalized as a recognizable diagnostic pathway. Neurovascular conflict language alone did not distinguish non-classical alveolar/post-dental syndromes from clean classical TN, emphasizing that imaging must be interpreted in the context of chronology and sensory examination. Pain occurring within a trigeminal distribution is not, by itself, sufficient to establish mechanism. Before labeling V2/V3-dominant or tooth-focused pain as classical TN, clinicians should explicitly address dental procedures, onset after extraction, lower-lip or chin numbness, intraoral sensory change, and failure of pain relief after dental intervention. Recognizing this phenotype early may reduce diagnostic delay and improve alignment between pain mechanism and treatment selection.

## Figures and Tables

**Table 1:** Clinical and dental-pathway characteristics by adjudicated phenotype.

Variable	A: Alveolar Neuropathy (n = 19)	B: Post-Extraction Onset (n = 31)	C: Odontogenic Diagnostic Misclassification (n = 20)	D: Mixed/Uncertain (n = 115)	E: Classical TN (n = 16)	*p* Value
Age, mean ± SD	52.8 ± 17.4	55.0 ± 17.1	56.6 ± 18.1	55.2 ± 15.7	57.1 ± 18.1	0.938
Female sex	11/19 (57.9%)	23/31 (74.2%)	13/20 (65.0%)	69/115 (60.0%)	8/16 (50.0%)	0.505
Explicit TN terminology	14/19 (73.7%)	17/31 (54.8%)	16/20 (80.0%)	54/115 (47.0%)	13/16 (81.2%)	0.005
V2 involvement	18/19 (94.7%)	28/31 (90.3%)	16/20 (80.0%)	90/115 (78.3%)	13/16 (81.2%)	0.318
V3 involvement	17/19 (89.5%)	27/31 (87.1%)	14/20 (70.0%)	82/115 (71.3%)	13/16 (81.2%)	0.197
Documented oral sensory deficit	6/19 (31.6%)	19/31 (61.3%)	2/20 (10.0%)	19/115 (16.5%)	0/16 (0.0%)	<0.001
Neuropathic wording in chart	15/19 (78.9%)	29/31 (93.5%)	5/20 (25.0%)	63/115 (54.8%)	7/16 (43.8%)	<0.001
Post-extraction onset	3/19 (15.8%)	24/31 (77.4%)	10/20 (50.0%)	4/115 (3.5%)	0/16 (0.0%)	<0.001 Descriptive only †
Any extraction documented	3/19 (15.8%)	20/31 (64.5%)	20/20 (100.0%)	8/115 (7.0%)	0/16 (0.0%)	<0.001
Any dental procedure	7/19 (36.8%)	26/31 (83.9%)	20/20 (100.0%)	40/115 (34.8%)	5/16 (31.2%)	<0.001
Dental evaluation before/within pathway	9/19 (47.4%)	18/31 (58.1%)	9/20 (45.0%)	91/115 (79.1%)	12/16 (75.0%)	0.002

**Note.** Values are n/N (%) unless otherwise indicated. Denominators reflect patients with evaluable documentation for each variable. Missing data were not imputed. Anatomical trigeminal-division variables refer only to explicitly documented branch-territory involvement. TN1/type 1 terminology was treated as TN phenotype terminology and was not coded as ophthalmic-division V1 involvement. A = confirmed alveolar neuropathy; B = post-extraction neuropathic onset; C = odontogenic diagnostic misclassification; D = mixed/uncertain dental-interface pain; E = clean classical TN. † Post-extraction onset contributed to phenotype adjudication, particularly for Group B, and was, therefore, presented descriptively only, rather than tested as an independent between-group variable.

**Table 3:** Imaging, medication exposure, and documented procedural exposure by adjudicated phenotype.

Variable	A (n = 19)	B (n = 31)	C (n = 20)	D (n = 115)	E (n = 16)	*p* Value
MRI documented	14/19 (73.7%)	29/31 (93.5%)	20/20 (100.0%)	96/115 (83.5%)	15/16 (93.8%)	0.067
Neurovascular conflict/vascular loop	7/19 (36.8%)	14/31 (45.2%)	6/20 (30.0%)	46/115 (40.0%)	6/16 (37.5%)	0.867
Multiple sclerosis	0/19 (0.0%)	4/31 (12.9%)	0/20 (0.0%)	20/115 (17.4%)	0/16 (0.0%)	0.030
Tumor-related disease	3/19 (15.8%)	5/31 (16.1%)	4/20 (20.0%)	32/115 (27.8%)	0/16 (0.0%)	0.095
Secondary structural disease	3/19 (15.8%)	9/31 (29.0%)	4/20 (20.0%)	52/115 (45.2%)	0/16 (0.0%)	<0.001
Carbamazepine exposure	16/19 (84.2%)	24/31 (77.4%)	19/20 (95.0%)	84/115 (73.0%)	15/16 (93.8%)	0.096
Pregabalin exposure	9/19 (47.4%)	17/31 (54.8%)	8/20 (40.0%)	53/115 (46.1%)	9/16 (56.2%)	0.791
Gabapentin exposure	2/19 (10.5%)	9/31 (29.0%)	4/20 (20.0%)	27/115 (23.5%)	7/16 (43.8%)	0.210
Oxcarbazepine exposure	6/19 (31.6%)	11/31 (35.5%)	6/20 (30.0%)	27/115 (23.5%)	1/16 (6.2%)	0.232
Any invasive procedure	19/19 (100.0%)	30/31 (96.8%)	13/20 (65.0%)	88/115 (76.5%)	15/16 (93.8%)	0.018
Blocks/injections	19/19 (100.0%)	30/31 (96.8%)	13/20 (65.0%)	88/115 (76.5%)	13/16 (81.2%)	0.006
Radiofrequency procedure	7/19 (36.8%)	15/31 (48.4%)	5/20 (25.0%)	35/115 (30.4%)	7/16 (43.8%)	0.293
Balloon compression	9/19 (47.4%)	6/31 (19.4%)	5/20 (25.0%)	16/115 (13.9%)	6/16 (37.5%)	0.006
Microvascular decompression	9/19 (47.4%)	14/31 (45.2%)	8/20 (40.0%)	19/115 (16.5%)	8/16 (50.0%)	<0.001
Stereotactic radiosurgery	4/19 (21.1%)	6/31 (19.4%)	2/20 (10.0%)	19/115 (16.5%)	6/16 (37.5%)	0.267

**Table 4:** Outcomes and treatment timing.

Variable	A (n = 19)	B (n = 31)	C (n = 20)	D (n = 115)	E (n = 16)	*p* Value
Any improvement documented	12/19 (63.2%)	28/31 (90.3%)	15/20 (75.0%)	90/115 (78.3%)	15/16 (93.8%)	0.102
Complete pain relief documented	7/19 (36.8%)	18/31 (58.1%)	9/20 (45.0%)	60/115 (52.2%)	9/16 (56.2%)	0.615
Recurrence documented	15/19 (78.9%)	27/31 (87.1%)	14/20 (70.0%)	82/115 (71.3%)	13/16 (81.2%)	0.402
First invasive treatment dated as performed	19/19 (100.0%)	28/31 (90.3%)	15/20 (75.0%)	92/115 (80.0%)	15/16 (93.8%)	0.082
Referral-to-first invasive treatment, median days [IQR]	0 [0–24]	8 [0–156]	0 [0–146]	0 [0–111]	40 [0–114]	0.269

**Note.** Timing represents first specialist documentation within the dataset to first invasive treatment explicitly charted as performed, not symptom onset to treatment.

## Data Availability

The data underlying this study are not publicly available due to institutional privacy restrictions and the presence of clinical free-text documentation.
